# Admissibility of Prior Sexual History Evidence: Examining Its Impact on Mock‐Jurors’ Judgments When Gender and Race Are Considered

**DOI:** 10.1002/bsl.70018

**Published:** 2025-10-10

**Authors:** Bailey M. Fraser, Emily Pica, Joanna D. Pozzulo

**Affiliations:** ^1^ Department of Psychology Carleton University Ottawa Ontario Canada; ^2^ Department of Psychological Science and Counseling Austin Peay State University Clarksville Tennessee USA

**Keywords:** adult victims, gender, mock juror decision making, prior sexual history, race, rape myths, sexual assault

## Abstract

Rape shield laws restrict the admission of prior sexual history evidence (PSHE) in sexual assault trials in various countries, including Canada and the U.S. Despite such laws, admission of PSHE is often at the discretion of a trial judge. The current study examined the effect of PSHE (present, absent), victim and defendant gender (male, female), and victim race (White, Indigenous) on mock‐juror decision‐making. Undergraduate students (*N* = 484) read a mock‐trial transcript depicting a rape case. Mock‐jurors provided guilt ratings and perceptions of the victim and defendant. Mock‐jurors assigned higher guilt ratings, held less favourable perceptions of the defendant, and more favourable perceptions of the victim, when PSHE was absent. Mock‐jurors also were more likely to reach a guilty verdict when the victim was male (as opposed to female). Finally, mock‐jurors perceived the defendant less favourably, and the victim more favourably, when the victim was Indigenous, as opposed to White.

## 1 Introduction

On June 22, 2011, an Indigenous woman named Cindy Gladue was found deceased in an Edmonton hotel room in Alberta, Canada (R. v. Barton 2017, 2019). The medical examiner in the case determined that Gladue bled to death from a large, blunt‐force to her vaginal area. The prosecution alleged that the defendant, Bradley Barton, sexually assaulted Gladue with a sharp object while she was drunk, aware that his actions would cause serious harm or death. However, Barton argued that Gladue's death was an accident. He testified at length about his previous sexual activity with Gladue, describing that he and Gladue engaged in similar consensual sexual activities on both the evening of her death and the evening prior.

The jury acquitted Barton of first‐degree murder, however, the Crown appealed for a new trial on the basis that Barton discussed Gladue's prior sexual activity without the evidence being admitted by the trial judge. It was argued that the trial judge wrongfully allowed Barton to discuss the victim's prior sexual history (under Section 276 of the Criminal Code of Canada), and did not provide instructions to the jury regarding this evidence, which may have swayed the jurors' perceptions of the victim. The Alberta Court of Appeal ordered a new trial for first‐degree murder; however, Barton appealed this decision. The case eventually reached the Supreme Court of Canada, and a new trial was ordered. This time, Barton was convicted for manslaughter (R. v. Barton 2019).

Many countries, including Canada (*Criminal Code*, RSC 1985, c. C—46, s 276[1]) and the United States (Federal Rules of Evidence, R. 412), have varying forms of ‘Rape Shield’ laws that restrict the use of evidence concerning a complainant's prior sexual history in sexual assault trials. Rape shield laws have been enacted to prevent the use of ‘twin myth’ reasoning, such that the defence may otherwise introduce a complainant's prior sexual activity as evidence to imply that a complainant is a) less believable or b) more likely to have consented to the sexual activity in question (see *Criminal Code*, 1985, s 276[1]; Dufraimont 2019; R. v. Seaboyer 1991). At the same time, it is important to note that although rape shield laws are aimed at protecting the rights of complainants, there exists some controversy, as excessive restriction of relevant evidence can threaten the accused's right to a fair trial.

Historically, the Criminal Code of Canada did not place specific restrictions on admission and use of prior sexual history evidence in sexual assault trials. However, in 1982, Canadian Parliament passed a ‘rape shield’ law (Bill C‐127) which officially became law in 1983 (Department of Justice Canada 1990); in the decades since, new rape shield statutes have been adopted and amended in Canada (McNabb and Baker 2021), with a similar history of rape shield law in the United States (Mueller et al. 2018; Wallach 1997). Although rape shield laws restrict the admission of a victim's prior sexual history, judges have discretion in determining admission of prior sexual history evidence (e.g., prior sexual history evidence may be admitted to provide context in cases where the victim and defendant had a prior relationship; see *Criminal Code*, 1985, s 276[1]). Moreover, the Criminal Code states that prior sexual history evidence may be admitted if ‘(a) it is relevant to an issue at trial (e.g., consent, identity, or credibility) and is not being used for the prohibited inferences described in Section 276(1)’, (b) ‘it is of specific instances of sexual activity’, and (c) ‘it has significant probative value that is not substantially outweighed by the potential prejudice to the complainant’. There have been a number of cases in which trial judges have erred in admitting prior sexual history evidence or have not intervened when such evidence was presented (e.g., R. v. Barton 2017). This can be problematic as the introduction of evidence regarding a victim's prior sexual history may influence how a victim is perceived. To our knowledge, there are no standard instructions that judges provide to jurors when prior sexual history evidence is admitted that could ameliorate jurors' biased perceptions.

Specifically, the admission of evidence regarding a victim's prior sexual history may favour the defendant and be detrimental to the victim's credibility (Schuller and Klippenstine 2004), as was likely the case in R. v. Barton (2017). Although research is limited, studies examining the impact of prior sexual history evidence have found that individuals presented with a complainant's prior sexual history perceive the complainant as less credible, more blameworthy, and are more likely to believe that the complainant consented to the sexual activity in question, than individuals who are not presented with this evidence (e.g., Herriott 2021; Monson et al. 2000; Schuller and Hastings 2002). Furthermore, research suggests that when the complainants and defendants had a prior sexual relationship, mock‐jurors viewed the case as less ‘clear‐cut’ than one involving stranger rape, leading to a lower likelihood of conviction due to assumptions about consent and resistance (Ellison and Munro 2013). These findings may be understood through dual‐system models of cognition, such that the presentation of prior sexual history evidence may prompt reliance on ‘System 1’ reasoning that involves fast, heuristic reasoning based on stereotypes of what constitutes ‘real rape’, rather than ‘System 2’ reasoning which is slower, more deliberate, and evidence‐based (Kahneman and Frederick 2005).

Although prior sexual history evidence has been identified as an important factor in sexual assault trials, research examining its impact on juror decision‐making remains limited. Further, there is no known research that has explored how prior sexual history evidence interacts with other factors, such as victim and defendant characteristics. Characteristics such as race and gender are particularly salient in sexual assault cases, with research suggesting that gender‐ and race‐based stereotypes influence mock‐jurors’ perceptions of the victim and defendant (McCoy and Gray 2007; Pozzulo et al. 2010; Wayne et al. 2001). Accordingly, there is a need to better understand how the admission of prior sexual history evidence, in combination with these characteristics, informs juror decision‐making.

### 1.1 Rape Myths

Although rates of police‐reported sexual assault have increased in recent years (e.g., a 38% increase between 2017 and 2022 in Canada; Conroy 2024), many cases that make it to prosecution do not result in a conviction (Dinos et al. 2015; Rotenberg 2017). Although there are many factors that influence whether a conviction occurs, it has been suggested that low conviction rates in sexual offence cases are in part due to ‘rape myths’ held by jurors (Shaw et al. 2017). Rape myths are incorrect beliefs regarding sexual misconduct, which typically place blame on the victim rather than the perpetrator. For example, a commonly held rape myth is that a victim ‘asked for it’ (Edwards et al. 2011).

Jurors may have misconceptions regarding sexual offences, and as such it is important to understand how rape myths influence mock‐juror decision‐making when relevant case factors are considered. Research indicates that mock‐jurors’ endorsement of rape myths influences perceptions and decisions in sexual offence cases. That is, mock‐jurors with high acceptance of rape myths typically perceive the victim's testimony as less credible and are less likely to find the defendant guilty (e.g., Dinos et al. 2015; Hammond et al. 2011; Pica et al. 2017). As research suggests that endorsement of rape myths is influential in sexual offence cases, the current research examined whether rape myth acceptance moderates the effects of prior sexual history evidence, victim and defendant gender, and victim race on mock‐jurors’ perceptions and decisions.

### 1.2 Defendant and Victim Gender

Gender is considered the primary factor in determining which groups are most vulnerable to sexual victimization, with the majority of sexual assault cases involving a female victim (Department of Justice Canada 2022). According to Statistics Canada, 30% of Canadian women have reported being victims of sexual assault since the age of 15, compared with only 8% of men (Cotter and Savage 2019). Further, in reported sexual assault cases in Canada, approximately 85% of victims are female, while 97% of perpetrators are male (Department of Justice Canada 2022), with similar rates in the United States (Department of Justice Canada 2022).

Although women represent the majority of victims in sexual assault cases, and men represent the majority of perpetrators of this assault, there also are instances in which men are victims to sexual assault (Choudhary et al. 2010; Stemple and Meyer 2014) and cases other than the ‘prototypical’ female victim and male perpetrator (e.g., male victim and male perpetrator). Further, surveys examining sexual orientation in sexual assault cases have found that compared with heterosexual individuals (women = 29.8%, male = 7.2%), lesbian/gay (women = 39.3%, men = 26.6%) and bisexual (women = 55.4%, male = 25.4%) individuals are more likely to be victims of sexual assault (Cotter and Savage 2019). However, portrayals of sexual victimization reinforce the prototype of a female victim and male perpetrator (MacKinnon 1989; Mardorossian 2014; Sivakumaran 2005), which is likely to be influential in a juror decision‐making context. Given the prevalence of sexual assault victimization among non‐heterosexual groups, it is particularly important to examine how jurors perceive alleged sexual assault that does not involve the prototypical male perpetrator and female victim dyadic.

In general, research has found that mock‐jurors perceive cases involving a female complainant and male defendant as more ‘prototypical’ of sexual assault than when the complainant is male, and the defendant is female (Starosta et al. 2025), these findings can be explained by social role theory which suggests that there are societal expectations for how men and women behave, based on stereotypes related to sex and gender (Eagly 1987). Research also suggests that male victims are typically perceived as more blameworthy than female victims (e.g., Gerber et al. 2004; Levi et al. 2024; Pica et al. 2021b; Sommer et al. 2016; Starosta and Schuller 2020), which could be because male victimization does not align with masculine stereotypes of sexual aggression and dominance (Murnen et al. 2002). However, other research has found no effect of victim gender (male vs. female) in sexual assault cases (Ellingwood et al. 2023; Pals et al. 2024), or the opposite effect, such that higher guilt ratings were found when the victim was male, as opposed to female (Pica et al. 2021a).

Research examining defendant gender has typically found that male defendants are perceived more negatively than female defendants. Moreover, mock‐juror research involving sexual misconduct cases have found that male defendants are perceived more negatively and are more likely to receive a guilty verdict than female defendants (e.g., Duke and Desforges 2007; Pozzulo et al. 2010; Quas et al. 2002; Stevens et al. 2022). However, some research has found that mock‐jurors are more likely to render a guilty verdict when the defendant is female, compared to male (e.g., Wayne et al. 2001). In a study examining both victim and defendant gender, Sommer et al. (2016) found that mock‐jurors had an increased likelihood of rendering a guilty verdict when the perpetrator was male and the victim was female, as opposed to when the perpetrator was female and the victim was male. Similarly, in the context of child sexual assault (CSA), Pals et al. (2022) found that participants perceived the CSA as more severe when the perpetrator was a male, as opposed to a female; however, there was no significant effect of victim gender.

In general, findings that mock‐jurors perceive male defendants of sexual misconduct more negatively than female defendants, and female victims more favourably than male victims, can be explained by gender‐role stereotypes and cognitive scripts. That is, mock‐jurors may use ‘rape scripts’ (cognitive scripts regarding what typically occurs during rape) to understand the sequence of events and the role of the victim and defendant in a sexual assault case (Peterson and Muehlenhard 2004), which may be based on rape myths regarding the possible gender of a defendant and victim. As such, mock‐jurors may perceive female victims more favourably, and male defendants more negatively, as the female victim‐male perpetrator dyadic is prototypical of sexual assault. Moreover, *non‐heterosexual* sexual misconduct may be perceived as more unwelcome by the victim than *heterosexual* sexual misconduct, as the behaviour does not reflect typical stereotypes of sexual assault.

Indeed, research examining the effect of same‐versus cross‐gender sexual misconduct has found that perpetrators in same‐gender cases were perceived more negatively (and were more likely to be convicted) than perpetrators in cross‐gender cases (Wayne et al. 2001). Other research has found that in cases of male‐perpetrated sexual assault, mock‐jurors held more pro‐victim perceptions when a male victim was straight as opposed to non‐heterosexual (Levi et al. 2024). Other research examining victim sexual orientation in a case involving a female victim and male perpetrator has found that mock‐jurors render more guilty verdicts and held more pro‐victim judgments when the victim stated she was lesbian, versus heterosexual or bisexual (Geoghagan et al. 2025).

While gender alone has been shown to influence juror decision‐making, it is important to recognize that victim and defendant gender may interact with other factors. As intersectionality theory suggests (Crenshaw 1989; Collins 2000), overlapping social identities, such as gender and race, interact to shape distinct social experiences. Accordingly, perceptions of victim credibility and defendant culpability may be influenced by multiple identities simultaneously, highlighting the importance of examining how the combination of race and gender impact juror outcomes in sexual assault cases.

### 1.3 Victim Race

Past research has typically found that victims are perceived more favourably, and defendants receive more punitive treatment, when a victim is White, as opposed to a racial minority (e.g., Baldus and Woodworth 2003; Bottoms et al. 2004; Mazzella and Feingold 1994; Pica et al. 2020; Williams et al. 2007). More recent research has challenged this pattern of findings, indicating that jurors may be more likely to render a guilty verdict when the victim is a racial minority, as opposed to White (e.g., Fraser et al. 2025; Huff et al. 2019). However, research examining victim race has typically compared White and Black victims, with few studies examining the effect of *Indigenous* victims of sexual assault. In a Canadian context, it is particularly important to understand how jurors perceive Indigenous victims due to Canada's colonial history (see Giannetta (2021) for a discussion) and a high prevalence of victimization among Indigenous Peoples (Statistics Canada 2022). Moreover, despite representing only 5% of the Canadian population (Statistics Canada 2022), 55% of women who reported being victim of sexual assault identified as Indigenous (First Nations, Métis, or Inuit), compared to 38% of women who identified as non‐Indigenous (Cotter and Savage 2019). Similarly, in the United States, Indigenous women (58%) report higher rates of lifetime sexual violence victimization than Indigenous men (28%), White women (49%), and White men (21%; Rosay 2016).

Stereotypical beliefs about Indigenous Peoples and racial bias can influence jurors' perceptions of victims of sexual assault. Indeed, research has indicated that Indigenous women are often perceived as being responsible for their victimization (Cormack and Balfour 2004). Findings from mock‐juror studies, however, are inconsistent. Some research indicates that Indigenous victims may elicit more punitive decisions towards defendants (e.g., ForsterLee et al. 2006; Fraser et al. 2025). Other studies, however, suggest the reverse. Pfeifer and Ogloff (2003) and Ewanation and Maeder (2023) both found that White victims were more likely to lead to guilty verdicts than Indigenous victims, though the latter examined police use of force rather than sexual assault. Overall, results regarding Indigenous victims remain mixed and appear to vary by crime type and study context. Given the limited and inconsistent findings, and the high prevalence of sexual victimization among Indigenous women in Canada, more research is warranted to understand how Indigenous identity shapes juror decision‐making in sexual assault cases.

Furthermore, these findings highlight the importance of examining not only the independent effects of gender and race, but also how they intersect. For instance, cultural narratives often position White women as ‘ideal victims’, while men of colour are stereotyped as ‘ideal perpetrators’ of crime (see Long 2021). When combined, these perceptions may disadvantage non‐White defendants in cases where the victim is White. Specifically, the sexual stratification hypothesis (LaFree 1989) suggests that perceptions of crime differ based on the race and gender of the defendant and victim, with sexual assault cases involving Black male defendants and White female victims resulting in harsher treatment of the defendant than other combinations of victim and defendant race and gender. Conversely, women of colour are often excluded from the cultural prototype of the ‘believable’ victim, which may lead jurors to discount their testimony or attribute greater responsibility to them for their victimization (Spohn and Tellis 2012). However, empirical research in mock‐juror settings has rarely explored how defendant and victim race and gender intersect in sexual assault cases. Addressing this gap is important for understanding disparities in trial outcomes and provides the basis for the current study.

### 1.4 The Current Research

The purpose of the current study was to examine the influence of prior sexual history evidence, victim and defendant gender, and victim race, on mock‐jurors’ decisions (i.e., dichotomous verdict, continuous guilt ratings, and perceptions of the victim's and defendant's testimony [e.g., reliability, honesty]) in a sexual assault case. The main hypotheses for the current study are as follows:

H 1 Main Effect of Prior Sexual History Evidence.

Although the literature is limited, it was predicted based on past research (e.g., Ellison and Munro 2013; Herriott 2021; Monson et al. 2000; Schuller and Hastings 2002) that mock‐jurors will (a) assign fewer guilty verdicts, (b) assign lower guilt ratings, (c) hold less pro‐victim judgments, and (d) hold more pro‐defendant judgments when prior sexual history evidence is presented, compared to when no prior sexual history evidence is presented.

H 2 Main Effect of Victim Gender.

Based on past research (e.g., Pica et al. 2021b; Sommer et al. 2016), it was hypothesized that mock‐jurors will (a) assign more guilty verdicts, (b) assign higher guilt ratings, (c) hold more pro‐victim judgments, and (d) hold less pro‐defendant judgments when the victim is female, as opposed to male.

H 3 Main Effect of Defendant Gender.

Based on previous studies (e.g., Duke and Desforges 2007; Pozzulo et al. 2010; Stevens et al. 2022), it was hypothesized that mock‐jurors will (a) assign more guilty verdicts, (b) assign higher guilt ratings, (c) hold more pro‐victim judgments, and (d) hold less pro‐defendant judgments when the defendant is male, as opposed to female.

H 4 Main Effect of Victim Race.

Based on recent research which has found that racial minorities are perceived more favourably than their White counterparts (e.g., Barager et al. 2025; Fraser et al. 2025; Huff et al. 2019), it was hypothesized that mock‐jurors will (a) assign more guilty verdicts, (b) assign higher guilt ratings, (c) hold more pro‐victim judgments, and (d) hold less pro‐defendant judgments when the victim is Indigenous, as opposed to White.

H 5 Exploratory Analyses.

Given the limited literature and inconsistencies in available research, we conducted several exploratory analyses. Specifically, we examined all two‐ and three‐way interactions between the predictor variables. We also explored whether participants' levels of rape myth acceptance moderated the extent to which prior sexual history evidence, victim and defendant gender, and victim race affected juror judgments. For example, individuals who strongly endorse rape myths might be more likely to discount the credibility of victims or to view defendants more favourably, particularly when prior sexual history evidence is presented.

## 2 Method

### 2.1 Participants

An a priori power analysis for a medium effect size (Cohen's *f* = 0.25) and 80% power was conducted using G*Power; the power analysis indicated that a sample size of 320 would be sufficient. Participants (*N* = 484) were recruited from a university in Eastern Ontario, Canada and screened for jury eligibility (i.e., 18‐year or older, a Canadian citizen, and have not been criminally convicted). The majority of participants were female (74.2%, *n* = 359) and the age of the sample ranged from 18 to 70 years (*M*_age_ = 20.33, SD = 4.68). The majority of the sample self‐identified as White (62.4%, *n* = 302), followed by Black (8.7%, *n* = 42), Mixed origin (7.0%, *n* = 34), West Asian (6.2%, *n* = 30), South Asian (5.0%, *n* = 24), East Asian (3.3%, *n* = 16), Indigenous (2.3%, *n* = 11), Southeast Asian (2.1%, *n* = 10), Latin American (1.7%, *n* = 8), and other (0.6%, *n* = 3); four participants (0.8%) chose not to disclose their ethnicity. All participants received course credit for taking part in the study.

### 2.2 Design

A 2 (prior sexual history evidence: present or absent) × 2 (victim gender: male or female) × 2 (defendant gender: male or female) × 2 (victim race: White or Indigenous) between‐subjects factorial design was used. The dependant variables included dichotomous verdict, continuous guilt ratings, and perceptions of the defendant and victim.

### 2.3 Measures

#### 2.3.1 Trial transcript

Sixteen versions of a seven‐page mock‐trial transcript that varied the presence of a victim's prior sexual history evidence (present, absent), victim gender (male, female), defendant gender (male, female), and victim race (White, Indigenous) were created.^1^ The transcript describes a sexual assault case in which the defendant (either male or female) was accused of sexually assaulting the victim (either male or female; White or Indigenous) while the two were at the victim's home. The trial transcript also describes that the victim and defendant either had a prior consensual sexual relationship or makes no mention of a prior sexual relationship. All other case details remained constant. The purpose of the prior sexual history manipulation was to examine the effect of *admitting* prior sexual history evidence in a trial, not to manipulate the actual existence of a prior relationship. In the ‘absent' condition, mock‐jurors were not told whether a prior sexual relationship existed, reflecting real‐world courtroom scenarios where such evidence may be excluded. In both conditions, the victim and defendant may or may not have had a prior relationship; the critical manipulation is whether mock‐jurors were exposed to information about prior sexual contact.

The mock‐trial transcript began with a case summary and general instructions for reading the transcript. Then mock‐jurors were provided instructions from the judge, followed by six witness testimonies (i.e., the police officer who took the victim's statement, the victim, a friend of the victim, a friend of the defendant, the defendant, and the defendant's coworker). The trial transcript concludes by providing closing statements from the Crown and Defence counsel and final instructions from the judge (see the trial transcript in Supporting Information S1).

#### 2.3.2 Verdict form

Mock‐jurors were instructed to rate the extent to which they believed the defendant was guilty on a 101‐point scale from 0 (not guilty) to 100 (guilty). Although continuous guilt ratings are not meaningful in a real‐life case (unlike dichotomous verdicts), continuous guilt ratings can help to understand mock‐jurors’ perceptions of defendant guilt when they are not forced to render a dichotomous guilt decision. After providing ratings of continuous guilt, mock‐jurors provided a dichotomous verdict (i.e., guilty or not guilty).

#### 2.3.3 Victim and defendant ratings

Participants rated their perceptions of the victim's testimony on a 101‐point scale (0 = *not at all*, 100 = *absolutely*), over several dimensions (e.g., truthfulness, accuracy, believability, honesty, accuracy, reliability, credibility). For example, mock‐jurors were asked, ‘how truthful do you believe the alleged victim to be’. Mock‐jurors also were asked to provide similar ratings in regard to the defendant's testimony. For example, mock‐jurors were asked, ‘How truthful do you believe the defendant to be’ on a scale from 0 [*not at all*] to 100 [*absolutely*].

#### 2.3.4 Updated Illinois Rape Myth Acceptance (IRMA) Scale

Mock‐jurors completed the 22‐item updated IRMA scale (McMahon and Farmer 2011) to determine acceptance of rape myths (e.g., ‘If a girl doesn't physically fight back, you can't really say it was rape’). Mock‐jurors rated their agreement with each statement on a scale from 1 (*strongly agree*) to 5 (*strongly disagree*). Lower scores indicated higher levels of endorsement of rape myths.

#### 2.3.5 Manipulation check

Participants were asked to complete four multiple‐choice questions relating to the manipulated variables to ensure that the trial transcript was read and understood. Specifically, mock‐jurors were asked to indicate whether the victim and defendant had a prior sexual relationship, the genders of the defendant and victim, and the race of the victim.

### 2.4 Procedure

Data were collected online using the survey tool Qualtrics. Participants completed the study individually as mock‐jurors. Each participant was assigned a unique study URL and was randomly assigned to one of the 16 conditions. Participants were instructed to read the mock‐trial transcript and then complete a series of questionnaires. Following this, participants completed the manipulation check questions and were debriefed and thanked for their participation.

## 3 Results

### 3.1 Manipulation Check

There were 612 participants who took part in the study. However, data from 128 individuals were excluded from further analyses because they failed one or more of the four manipulation check questions. Each manipulation check was designed to ensure that participants were aware of the experimental conditions: one question asked whether prior sexual history evidence was presented, one question asked about the defendant's gender, one question asked about the victim's gender, and one question asked about the victim's race. Data from the remaining 484 participants were included in the following analyses.

### 3.2 Dichotomous Verdict (Hypotheses 1a, 2a, 3a, and 4a)

Participants rendered a dichotomous verdict decision based on the trial transcript they were asked to read. A hierarchical logistic regression analysis was conducted to examine the effect of prior sexual history evidence, defendant gender, victim gender, and victim race on mock‐jurors’ dichotomous verdict decisions (0 = *not guilty*, 1 = *guilty*). Mock‐juror characteristics (gender and race) were included as controls in the model; however, the addition of mock‐juror race did not influence results or improve model fit, thus only mock‐juror gender was included in the final model.^2^ Model one included mock‐juror gender, Model 2 contained all main effects, Model 3 included the two‐way interaction terms, and Model 4 included the three‐way interaction terms.

While Blocks 1 and 2 significantly improved model fit, the addition of Blocks 3 and 4 did not result in a significant improvement. Therefore, Model 2 was retained, *χ* ^2^ (5) = 36.77, *p* < 0.001. Controlling for mock‐juror gender, there was found to be a significant effect of victim race, such that the odds of rendering a guilty verdict were 1.5 times higher when the victim was Indigenous, as opposed to White, *b* = 0.41, SE = 0.19, Wald's *χ* ^2^ (1) = 4.41, *p* = 0.036, Exp(b) = 1.50, 95% CI [1.03, 2.20]. There were no significant effects of prior sexual history evidence (*p* = 0.07), victim gender (*p* = 0.07), or defendant gender (*p* = 0.42). See Table 1 for proportion of guilty verdicts by prior sexual history evidence, defendant and victim gender, and victim race.^3^

**TABLE 1:** Proportion of guilty verdicts by prior sexual history evidence (PSHE), defendant and victim gender, and victim race.

		Proportion guilty
PSHE	Present	0.55
	Absent	0.64
Victim gender	Male	0.64
	Female	0.55
Defendant gender	Male	0.61
	Female	0.58
Victim race	Indigenous	0.64
	White	0.55

#### 3.2.1 Dichotomous Verdict and IRMA

Participants responded to the updated Illinois Rape Myth Acceptance (IRMA; McMahon and Farmer 2011) scale which consists of 22 items that examine participants' acceptance of various rape myths. The 22 items were significantly correlated (*p* < 0.001) and the overall reliability of the scale was 0.93. Thus, a composite score was created by summing responses for the 22 items, with higher scores indicating greater *rejection* of rape myths. Overall, the sample held high rejection of rape myths (*M* = 96.56, SD = 13.03), with scores ranging from 27 to 110.

An exploratory hierarchical binary logistic regression analysis was conducted to determine whether mock‐jurors’ degree of rape myth acceptance (i.e., composite IRMA score^4^) moderated the effect of the predictor variables (prior sexual history evidence [PSHE], victim and defendant gender, and victim race) on dichotomous verdict. Model 1 contained mock‐juror gender, Model 2 contained the moderator variable (IRMA) and the predictor variables, and Model 3 contained the two‐way interaction terms between IRMA and the predictor variables. Although Blocks 1 and 2 were significant, Block 3 which represented the addition of the interaction terms was not significant (*p* = 0.12). However, we retained Model 3 as it was significant overall, *χ* ^2^ (10) = 46.49, *p* < 0.001, and we were interested in exploring the moderating effects of IRMA.

Controlling for mock‐juror gender, there was a significant interaction between IRMA and PSHE, *b* = 0.04, SE = 0.02, Wald's *χ* ^2^ (1) = 4.80, *p* = 0.028, Exp(b) = 1.04, 95% CI [1.00, 1.07]. To interpret this interaction, the simple slopes of PSHE were examined at high (+1 SD above the mean), moderate, and low (−1 SD below the mean) levels of rape myth rejection.^5^ At high levels of rape myth rejection, the presence of PSH did not influence verdict, *b* = −0.08, SE = 0.27, Wald's *χ* ^2^ (1) = 0.08, *p* = 0.78, Exp(b) = 0.93 (95% CI [0.55, 1.58]). Likewise, at moderate levels of rape myth rejection, prior sexual history evidence did not significantly predict dichotomous verdicts, *b* = 0.34, SE = 0.19, Wald *χ* ^2^ (1) = 3.17, *p* = 0.08, Exp(b) = 1.40 (95% CI [0.97, 2.02]). However, at low levels of rape myth rejection, prior sexual history evidence significantly predicted verdicts, such that mock‐jurors were 2.1 times more likely to render a guilty verdict when prior sexual history evidence was absent, as opposed to present, *b* = .75, SE = .27, Wald's *χ* ^2^ (1) = 7.49, *p* = 0.006, Exp(b) = 2.11, 1.24, 3.60]. IRMA was not found to significantly moderate the effects of the remaining predictor variables.

### 3.3 Continuous Guilt (Hypotheses 1b, 2b, 3b, and 4b)

A four‐way ANCOVA was conducted to determine the effects of prior sexual history evidence, defendant gender, victim gender, and victim race on mock‐jurors’ continuous guilt ratings (see Table 2 for descriptive statistics), controlling for mock‐juror gender. There was a significant main effect of prior sexual history evidence, *F* (1, 467) = 8.88, *p* = 0.003, partial *η* ^2^ = 0.02. Mock‐jurors assigned higher guilt ratings to the defendant when prior sexual history evidence was absent (*M* = 66.03, SE = 1.65) compared to when prior sexual history evidence was presented (*M* = 59.30, SE *= 1.55*). There also was a significant effect of victim race, *F* (1, 467) = 4.11, *p* = 0.04, partial *η* ^2^ = 0.01. Mock‐jurors assigned higher guilt ratings when the victim was Indigenous (*M* = 64.96, SE = 1.64), as opposed to White (*M* = 60.38, SE = 1.56). There were no significant effects of defendant gender or victim gender, and no significant interaction effects.

**TABLE 2:** Mean continuous guilt ratings (SD) by prior sexual history evidence (PSHE), defendant and victim gender, and victim race.

	PSHE absent	*n*	PSHE present	*n*
Male defendant				
Indigenous, male victim	67.92 (24.93)	26	66.28 (26.28)	32
White, male victim	66.07 (22.25)	30	61.35 (20.77)	34
Indigenous, female victim	73.11 (22.12)	28	57.38 (31.36)	29
White, female victim	66.55 (22.05)	29	54.81 (21.72)	31
Female defendant				
Indigenous, male victim	65.37 (29.46)	24	63.19 (26.62)	31
White, male victim	66.70 (21.00)	33	61.71 (26.70)	35
Indigenous, female victim	72.14 (18.76)	29	53.28 (28.45)	32
White, female victim	51.10 (24.94)	29	55.50 (31.05)	32

#### 3.3.1 Continuous Guilt and IRMA

An exploratory hierarchical regression analysis was conducted to examine whether mock‐jurors’ responses on the IRMA scale moderated the effects of the predictor variables (PSHE, defendant gender, victim gender, and victim race) on continuous guilt, controlling for mock‐juror gender. Model 1 contained mock‐juror gender, Model 2 contained IRMA (mean‐centred) and the predictor variables, and Model 3 contained the two‐way interaction terms between IRMA and the predictor variables. Model 3 did not significantly improve model fit, Δ*R* ^2^ = 0.005, *p* = 0.58, and there were no significant interactions between IRMA and the predictor variables. This suggests that mock‐jurors’ rape myth endorsement did not moderate the effects of prior sexual history evidence, defendant gender, victim gender, or victim race on continuous guilt ratings.

### 3.4 Defendant Perception Ratings (Hypotheses 1c, 2c, 3c, and 4c)

Participants were asked a series of questions regarding their perceptions of the defendant (i.e., responsibility for crime, believability, credibility, honesty, reliability, truthfulness, control over situation^6^). All seven items were significantly correlated (*p* < 0.001), and thus a composite score was created (*α* = 0.91) by averaging participant responses across all questions; higher scores indicate more favourable perceptions of the defendant.

A four‐way ANCOVA was conducted to examine the effect of prior sexual history evidence, defendant gender, victim gender, and victim race on mock‐jurors’ perceptions of the defendant, controlling for mock‐juror gender (see Table 3 for descriptive statistics). There was a significant effect of prior sexual history evidence, *F* (1, 467) = 4.02, *p* = 0.046, partial *η* ^2^ = 0.01. Mock‐jurors held more favourable perceptions of the defendant when prior sexual history evidence was present (*M* = 41.53, SE = 1.23), as opposed to when prior sexual history evidence was absent (*M* = 37.93, SE = 1.31). There also was a significant effect of defendant gender, *F* (1, 467) = 10.44, *p* = 0.001, partial *η* ^2^ = 0.02, such that mock‐jurors held more favourable perceptions of the defendant when the defendant was female (*M* = 42.64, SE = 1.27), as opposed to male (*M* = 36.83, SE = 1.28). When examining victim race, there was a significant main effect, *F* (1, 467) = 9.34, *p* = 0.002, partial *η* ^2^ = 0.02, such that mock‐jurors held more favourable perceptions of the defendant when the victim was White (*M* = 42.48, SE = 1.24), than when the victim was Indigenous (*M* = 36.99, SE = 1.30). There was no significant effect of victim gender.

**TABLE 3:** Mean defendant perception ratings (SD) by prior sexual history evidence (PSHE), defendant and victim gender, and victim race.

	PSHE absent	*n*	PSHE present	*n*
Male defendant				
Indigenous, male victim	37.23 (20.75)	26	37.21 (19.92)	32
White, male victim	37.19 (18.61)	30	37.62 (19.29)	34
Indigenous, female victim	27.93 (16.91)	28	35.87 (17.02)	29
White, female victim	38.00 (23.17)	29	45.24 (18.80)	31
Female defendant				
Indigenous, male victim	40.55 (23.19)	24	38.90 (18.03)	31
White, male victim	38.94 (19.04)	33	43.67 (19.53)	35
Indigenous, female victim	33.81 (21.20)	29	45.36 (23.75)	32
White, female victim	49.12 (18.21)	29	49.30 (23.74)	32

*Note:* Higher scores indicate more favourable perceptions of the defendant.

There was a significant two‐way interaction between victim gender and victim race, *F* (1, 467) = 4.59, *p* = 0.03, partial *η* ^2^ = 0.01. The interaction was followed‐up be examining pairwise comparisons; a Bonferroni correction was used to account for multiple comparisons. There was a significant simple main effect such that when the victim was female, mock‐jurors held more favourable perceptions of the defendant when the victim was White (*M* = 45.42) as opposed to Indigenous (*M* = 35.74; *M*_Diff_ = 9.67, SE = 2.61, *p* < 0.001, 95% CI [4.54, 14.81]; however, there was no difference in perception ratings for White vs. Indigenous victims when the victim was male. The remaining interactions were not significant.

#### 3.4.1 Defendant Perception Ratings and IRMA

An exploratory hierarchical regression analysis was conducted to examine whether mock‐jurors’ responses on the IRMA scale moderated the effects of the predictor variables (prior sexual history evidence, defendant gender, victim gender, and victim race) on defendant perception ratings, controlling for mock‐juror gender. Model 1 contained mock‐juror gender, Model 2 contained the main effects of IRMA and the predictor variables, and Model 3 contained the two‐way interaction terms between IRMA and the predictor variables. Model 3 did not significantly improve model fit, Δ*R* ^2^ = 0.01, *p* = 0.24, and there were no significant interactions between IRMA and the predictor variables. This suggests that mock‐jurors’ rape myth endorsement did not moderate the effects of prior sexual history evidence, defendant gender, victim gender, or victim race on defendant perception ratings.

### 3.5 Victim Perception Ratings (Hypotheses 1d, 2d, 3d, and 4d)

Similar to defendant perception ratings, participants were asked a series of questions regarding their perceptions of the victim (i.e., truthfulness, accuracy, honesty, believability, trustworthiness, reliability, and responsibility^7^). All seven items were significantly correlated (*p* < 0.001) and a composite score was created (*α* = 0.95) by averaging responses across all items; higher scores indicate more favourable perceptions of the victim.

A four‐way ANCOVA was conducted to examine the effect of prior sexual history evidence, defendant gender, victim gender, and victim race on mock‐jurors’ perceptions of the victim, controlling for mock‐juror gender (see Table 4 for descriptive statistics). There was a significant effect of prior sexual history evidence, *F* (1, 467) = 7.96, *p* = 0.005, partial *η* ^2^ = 0.02. Mock‐jurors held more favourable perceptions of the victim when prior sexual history evidence was absent (*M* = 69.28, SE = 1.48), compared to when prior sexual history evidence was presented (*M* = 63.56, SE = 1.39). There also was a significant effect of victim gender, *F* (1, 467) = 4.27, *p* = 0.039, partial *η* ^2^ = 0.01; mock‐jurors held more favourable perceptions of the victim when the victim was male (*M* = 68.52, SE = 1.43), as opposed to female (*M* = 64.32, SE = 1.44). When examining victim race, there also was a significant main effect, *F* (1, 466) = 12.53, *p* < 0.001, partial *η* ^2^ = 0.03; mock‐jurors held more favourable perceptions of the victim when the victim was Indigenous (*M* = 70.01, SE = 1.47), than when the victim was White (*M* = 62.83, SE = 1.40). There was no significant effect of defendant gender, and none of the interactions were significant.

**TABLE 4:** Mean victim perception ratings (SD) by prior sexual history evidence (PSHE), defendant and victim gender, and victim race.

	PSHE absent	*n*	PSHE present	*n*
Male defendant				
Indigenous, male victim	70.27 (21.77)	26	69.75 (24.35)	32
White, male victim	73.43 (18.81)	30	62.27 (24.45)	34
Indigenous, female victim	76.47 (16.27)	28	68.34 (26.42)	29
White, female victim	65.07 (23.76)	29	57.46 (19.56)	31
Female defendant				
Indigenous, male victim	68.41 (27.51)	24	71.67 (24.18)	31
White, male victim	69.25 (23.72)	33	65.71 (20.69)	35
Indigenous, female victim	74.93 (19.29)	29	59.13 (24.51)	32
White, female victim	57.22 (24.08)	29	53.13 (22.92)	32

#### 3.5.1 Victim Perception Ratings and IRMA

An exploratory hierarchical regression analysis was conducted to examine whether mock‐jurors’ responses on the IRMA scale moderated the effects of the predictor variables (prior sexual history evidence, defendant gender, victim gender, or victim race) on victim perception ratings, controlling for mock‐juror gender. Model 1 contained mock‐juror gender. Model 2 contained the main effects for IRMA and the predictor variables, and Model 3 contained the two‐way interaction terms between IRMA and the predictor variables. Model 3 did not significantly improve model fit, Δ*R* ^2^ = 0.005, *p* = 0.57, and there were no significant interactions between IRMA and the predictor variables. This suggests that mock‐jurors’ rape myth endorsement did not moderate the effects of prior sexual history evidence, defendant gender, victim gender, or victim race on victim perception ratings.

## 4 Discussion

The purpose of the current study was to examine the influence of the admission of a victim's prior sexual history evidence, defendant and victim gender, and victim race on mock‐jurors’ perceptions and decisions in a sexual assault case. Admission of prior sexual activity evidence is often at the discretion of a trial judge in Canada (see *Criminal Code*, 1985, s 276[1]; Dufraimont 2019). Importantly, admission of prior sexual history evidence may be detrimental to a victim's credibility (Schuller and Klippenstine 2004), as was likely the case in R. v. Barton (2017); at the same time, excessive restriction of relevant evidence can impede a defendant's right to a fair trial. Therefore, it is important to understand how admission of a victim's prior sexual history, in combination with relevant factors such as gender and race, influences mock‐jurors’ decision‐making. The current study found that admission of prior sexual history evidence (PSHE) influenced mock‐jurors’ perceptions and decisions. Moreover, although PSHE did not significantly influence binary verdict decisions (i.e., guilty vs. not guilty), rape myths significantly moderated the effect of PSHE on binary verdicts. Additionally, defendant gender, victim gender, and victim race influenced continuous measures; however, these variables did not impact binary verdicts. Together, these findings suggest that while biases and attitudes shape jurors' perceptions, they may not override the high evidentiary threshold required for criminal convictions.

### 4.1 Prior Sexual History Evidence

As hypothesized, the current research found that mock‐jurors assigned lower guilt ratings, held more favourable perceptions of the defendant, and less favourable perceptions of the victim when the victim's prior sexual history evidence (PSHE) was presented, compared to when PSHE was absent. These findings suggest that when a victim's prior sexual history evidence is admitted, mock‐jurors may perceive the victim as less believable or more likely to have consented to the sexual activity in question. However, while PSHE influenced continuous measures (i.e., continuous guilt, defendant perceptions ratings, and victim perceptions ratings), the effect of PSHE did not extend to dichotomous verdict decisions. One plausible explanation for this lies in the cognitive demands and decisional thresholds associated with different types of judgments. Continuous measures allow mock‐jurors to express more nuanced attitudes, which may be more sensitive to potentially prejudicial information (e.g., prior sexual history evidence), than binary verdicts which require a firm evidentiary threshold. In criminal trials, jurors are required to find a defendant guilty ‘beyond a reasonable doubt’, a high standard that may override personal biases or heuristic judgments when rendering a verdict. Thus, while PSHE may shift attitudes and perceptions (reflected in continuous ratings), it may not be sufficient to meet the threshold required for a guilty verdict. Moreover, compared with continuous ratings, verdict decisions likely involve more deliberative processing and are constrained by mock‐jurors’ understanding of the legal criteria required for conviction, possibly serving as a buffer against the influence of prejudicial information. These findings highlight the importance of examining both juror perceptions and formal verdict outcomes to fully understand the implications of admitting PSHE in court.

Although PSHE did not influence verdict decisions, an exploratory analysis found that mock‐jurors’ endorsement of rape myths moderated the effect of PSHE on dichotomous verdict decisions. That is, mock‐jurors who held high endorsement of rape myths were more likely to render a guilty verdict when PSHE was absent than when PSHE was presented; there was no significant effect of PSHE on dichotomous verdicts among mock‐jurors with moderate and low rape myth endorsement. Participants' rape myth acceptance was not found to significantly moderate the effect of admission of PSHE on the remaining dependent variables. It could be that individuals high in rape myth endorsement may rely more heavily on heuristic reasoning when presented with prior sexual history evidence, interpreting it as indicative of victim consent or reduced credibility. In contrast, individuals lower in rape myth endorsement may be more likely to engage in evidence‐based reasoning, discounting the relevance of such information. These results indicate the importance of considering jurors' pre‐existing beliefs in sexual assault cases, highlighting potential opportunities for juror screening and judicial instructions.

### 4.2 Defendant and Victim Gender

Contrary to hypotheses, there was no effect of defendant gender on dichotomous verdict decisions, continuous guilt ratings, or perceptions of the victim. However, mock‐jurors held more favourable perceptions of the defendant when the defendant was female, as opposed to male. This finding is consistent with past research (e.g., Duke and Desforges 2007; Mazzella and Feingold 1994; Stevens et al. 2022). As mentioned previously, this may be in part due to gender role stereotypes and the prototypical portrayal of sexual assault involving a male perpetrator and female victim. Lev‐Wiesel (2004) suggests that this is related to rape myths concerning the prototypical sexual assault and that the further the sexual assault deviates from this prototypical male‐female dyad, the less likely it is for them to find the defendant guilty.

When examining victim gender, mock‐jurors were found to hold more favourable perceptions of the victim when victim was male, as opposed to female. There was no significant effect of victim gender on dichotomous verdict, continuous guilt ratings, or perceptions of the defendant. The current findings are in contrast with past research that has typically found that male victims are perceived more negatively than female victims (e.g., Gerber et al. 2004; Pica et al. 2021b; Sommer et al. 2016; Starosta and Schuller 2020). However, some research has found that mock‐jurors perceive female victims more negatively than male victims (e.g., Pica et al. 2021a). Mock‐jurors may have perceived male victims more favourably than female victims due to the possibility of increased awareness of male victimization. Although exploratory analyses were conducted to examine whether defendant and victim gender had an interaction effect on the outcome variables, no such interactions were found. These findings suggest that stereotypes regarding gender may be shifting, and that greater awareness of male victimization could influence perceptions in sexual assault cases.

### 4.3 Victim Race

The current research found that mock‐jurors were more likely to render a guilty verdict, assigned higher guilt ratings to the defendant, held less favourable perceptions of the defendant, and held more favourable perceptions of the victim when the victim was Indigenous, as opposed to White. Victim race also was found to significantly interact with victim gender when examining mock‐jurors’ perceptions of the defendant. That is, when the victim was female, mock‐jurors held more favourable perceptions of the defendant when the victim was White, compared to Indigenous; there was no difference in perception ratings when the victim was male.

The current findings are consistent with recent mock‐juror research which has found that racial minorities are perceived more positively than White individuals (e.g., Barager et al. 2025; Fraser et al. 2025; Stone 2023). However, the findings are in contrast with earlier studies finding that victims of racial minorities are perceived more negatively than White victims (e.g., Baldus and Woodworth 2003; Mazzella and Feingold 1994; Pica et al. 2020; Williams et al. 2007). Research has suggested that recent trends of lower conviction rates for racial minority defendants in mock‐juror research could be due to social desirability bias, such that participants do not want to appear racially biased (e.g., Salerno et al. 2023; Smalarz et al. 2023); it is possible that social desirability effects extend to victim race. In addition, the current findings could indicate greater awareness of racial issues involving Indigenous Peoples, resulting in more favourable perceptions of Indigenous victims. For example, recent news coverage has highlighted racial issues affecting Indigenous peoples in Canada, such as the crisis of Missing and Murdered Indigenous Women and Girls (MMIWG; Stefanovich 2024) and the discovery of unmarked graves at former residential school sites across Canada (Deer 2024). Regardless, it is important to note that majority of participants in the current sample were White (62%), and therefore, findings may not be representative of non‐White mock‐jurors.

### 4.4 Limitations and Future Directions

The current research has certain limitations typical of mock‐juror studies, particularly concerning its ecological validity. Willmott et al. (2021) outline several methodological standards of mock‐juror research to maintain ecological validity. At minimum, Willmott and colleagues propose that participants should be considered jury eligible; the current research meets this standard as participants were jury eligible in Canada (aged 18‐year or older, a Canadian citizen, and not previously convicted of a criminal conviction).

Secondly, Willmot and colleagues suggest that participants are selected from a databased that summons real‐life jurors. The current study is limited in this aspect as a student sample was used. This limits ecological validity as it is possible that students' perceptions may differ from the general public (from which jurors are selected; Curley and Peddie 2024). However, it is important to note that Bornstein et al. (2017) found negligible differences between student and community samples across both criminal and civil mock‐juror studies.

Third, Willmott et al. propose that mock‐juror research should include jury deliberation. The current study examined individual juror decisions as opposed to jury deliberation decisions. However, researchers have found that, at best, 15% of individual jurors change their mind during deliberation (Booth et al. 2017). In addition, the current research does inform on pre‐deliberation biases. Incorporating a deliberation component into mock‐juror studies can enhance ecological validity (Curley and Peddie 2024; Nuñez et al. 2011); however, this addition may also compromise internal validity. As noted by Curley and Peddie (2024), the deliberation process can introduce confounding factors, such as the personal characteristics of jurors.

The fourth standard suggested by Willmott and colleagues is that mock‐juror studies ought to use realistic trial materials, the fifth standard is that studies should employ legal professionals to ensure that mock‐evidence would be admissible in a real‐life case, and the sixth standard is that research should replicate real‐life trials as realistically as possible. The current research is limited in regard to these three standards such that mock‐jurors were asked to read a written mock‐trial transcript and legal professionals were not consulted. However, despite these limitations, the materials used in the current research were realistic such that they involved opening and closing statements from the Crown and Defence, statements from six witnesses (who were cross‐examined), and laws from the Criminal Code of Canada that were relevant to the case. In addition, the trial transcripts used in the current research were realistic such that they were lengthy at approximately 3000 words.

The current study highlights several considerations for future research. In general, future juror research should aim to improve ecological validity by meeting the standards outlined by Willmott and colleagues. In addition to methodological limitations, certain demographic characteristics of the current sample also limit the generalisability of findings. Specifically, 74.2% of participants identified as female, 62.4% identified as White, and all were university students. Although student samples are common in mock‐juror research and prior studies have found minimal differences between student and community samples (Bornstein et al. 2017), the overrepresentation of certain demographic groups may still influence findings. Future research should examine the impact of *mock‐juror* characteristics such as gender, race, and educational level on rape myth acceptance and juror decision‐making. This is an important area of research because mock‐jurors have been found to differ in their perceptions and decisions based on demographic characteristics. For example, men and women have been shown to differ in their levels of rape myth endorsement (Belyea and Blais 2023; Byrne et al. 2021).

In addition, given shifting trends in race‐related effects, future mock‐juror research examining race should include a measure assessing social desirability bias to better account for potential biases in participants' responding. Including a measure of social desirability would help to determine whether findings reflect participants' genuine attitudes or attempts to respond in socially acceptable ways, improving the validity of race‐related effects. Further, although this study examined the effect of victim race, it did not vary defendant race. Future research could explore how the race of the defendant interacts with that of the victim. Additionally, future research should continue to examine the impact of a victim's prior sexual history in different contexts and with other relevant variables (e.g., juror instructions regarding prior sexual history evidence). This would identify the contexts in which prior sexual history evidence may be particularly influential and could inform guidelines for its admissibility in court. Finally, to better understand the divergence between perceptual judgments (e.g., credibility, guilt ratings) and binary verdicts, future studies could include questions probing participants' individual reasoning and decision thresholds. These open‐ended responses may provide insight into how jurors reconcile personal beliefs with legal standards.

### 4.5 Conclusion

This research is important as few mock‐juror studies have examined the impact of prior sexual history evidence, in combination with race and gender. The current study suggests that admission of prior sexual history evidence, as well as victim gender and victim race, are influential in the context of a mock‐juror sexual assault case. Although prior sexual history evidence did not significantly interact with race or gender variables, an interaction between victim gender and race was observed, indicating that victim characteristics play a meaningful role in shaping juror perceptions in sexual assault cases. In addition, the pervasive nature of rape myths surrounding prior sexual history was found to influence mock‐jurors’ dichotomous guilt which is troublesome as an individual's prior sexual history could be influential during a case involving sexual assault. These results have important implications for juror screening, judicial instructions, and admissibility of prior sexual history evidence, and highlight an opportunity to educate prospective jurors on non‐judicial factors that can impact decision‐making.

## Author Contributions

B.M.F. contributed to the study design, conducted data analyses, interpreted data, and drafted the manuscript. E.P. contributed to the study design and provided critical contributions to the manuscript. J.D.P. contributed to the study design and provided critical contributions to the manuscript.

## Ethics Statement

Ethical approval was received from Carleton University's Research Ethics Board B (CUREB‐B # 112176).

## Consent

All participants provided informed consent prior to participating in the study.

## Conflicts of Interest

The authors declare no conflicts of interest.

## Supporting information


Supporting Information S1

## Data Availability

The data that support the findings of this study are available from the corresponding author upon reasonable request.

## Endnotes

^1^
Each trial transcript was approximately 3000 words.

^2^
This also is true for all remaining analyses.

^3^
Overall, 59% of mock‐jurors rendered a guilty verdict.

^4^
IRMA scores were mean‐centred.

^5^
This technique is recommended by Cohen et al. (2003).

^6^
The items ‘*responsibility for crime’* and ‘*control over situation’* were reverse scored.

^7^
The item ‘*responsibility for what happened*’ was reverse scored.
