# Supplementary material for: Admissibility of Prior Sexual History Evidence: Examining Its Impact on Mock‐Jurors’ Judgments When Gender and Race Are Considered
Source: Behav Sci Law. 2025 Oct 10;44(1):20–33. doi: 10.1002/bsl.70018 (PMC12865671; doi:10.1002/bsl.70018)
Supplement: Supplementary file 1 — Supporting Information S1 [file BSL-44-20-s001.docx]

**Trial Transcript**

Taylor Dunn is a 28-year-old **man/woman** who has been charged with the sexual assault of **Mr./Ms.** Casey Erickson. Casey Erickson, **an Indigenous/a White** **man/woman,** reported the sexual assault to the Ottawa police, stating that **Mr./Ms.** Dunn had sexually assaulted **her/him** on the evening of October 17. **It is noted throughout the trial that Casey Erickson and Taylor Dunn had an ongoing, sexual relationship.** The following are excerpts taken from the transcript of Taylor Dunn’s criminal trial. Please read through the trial transcript as if you were one of the jurors tasked with having to make a decision on this case. Once you have finished reading the trial transcript you will be asked to determine a verdict of guilty or not guilty as well as answer some follow up questions on your opinions of trial witnesses and the defendant.

**Please read through the case carefully because you will not be able to refer back to the transcript when responding to some of the questions.**

**Judge:** Taylor Dunn has been charged with section 271 of the Criminal Code, sexual assault. Under Canadian Law, the Crown has the burden of proving that the defendant is guilty of this charge beyond a reasonable doubt. It is your responsibility to listen to all the evidence presented in this case, to decide the facts, and then to apply the law that I will give to you at the end of this trial. This case will begin with the Crown and Defence presenting their opening statements. These statements are summaries of what will be presented throughout the trial and are not evidence. Each attorney will then present and question witnesses and law enforcement personnel, who will subsequently be cross-examined. Please listen to the following proceedings carefully. Following the testimonies, you will be asked to decide as to whether the defendant, Taylor Dunn, is guilty or not guilty.

*The Crown makes their opening statement*.

**Crown:** **Mr./Ms.** Dunn sexually assaulted my client, **Mr./Ms.** Erickson on the evening of October 17, 2015. The evening that the sexual assault took place, **Mr./Ms.** Dunn had invited **Mr./Ms.** Erickson over to **his/her** apartment. The two of them ordered takeout for dinner and then watched some TV on the couch. While watching TV, **Mr./Ms.** Dunn saw an opportunity to take advantage of Casey Erickson, a young **Indigenous/White man/woman,** and proceeded to make inappropriate and unwanted sexual advances toward **him/her**. As a consequence of **his/her** actions, it is your duty as representatives of the community to seek justice by finding **Mr./Ms.** Dunn guilty of sexual assault.

*The Defence makes their opening statement*.

**Defence:** My client, **Mr./Ms.** Dunn, did not sexually assault Casey Erickson. Yes, it is true that my client was with **Mr./Ms.** Erickson on the night of October 17. However, the events of that night did not unfold the way in which the Crown wants you to believe. **Mr./Ms.** Dunn and **Mr./Ms.** Erickson had **consensual sex** **with each other numerous times before and had both consented to having sexual intercourse the evening of October 17**/ **had both consented to having sexual intercourse the evening of October 17**. It wasn’t until the following morning that **Mr./Ms.** Erickson decided that the previous night’s sexual interaction was unwanted. I trust that you will consider all this information accordingly and find my client, **Mr./Ms.** Dunn, is not guilty of sexual assault.

The Crown calls their first witness, Jonathan Schwab, and he takes the stand.

**Crown:**Please state your name and how you know the victim.

**Witness:**My name is Jonathan Schwab, and I am the police officer who spoke with **Mr./Ms.** Erickson the day that **he/she** came to the police station to report the sexual assault.

**Crown:**Thank you Mr. Schwab. Can you please explain to me the interaction between you and Casey Erickson when **he/she** brought the information of the assault to you?

**Witness: Certainly. Casey came in saying that he/she had been with Mr./Ms. Dunn the night before and that Mr./Ms. Dunn had sexually assaulted him/her.**

**Crown: Did you find Casey’s story credible?**

**Witness: Yes, I did. His/Her statement had no holes in it, and the emotions he/she displayed while discussing the incident could not have been faked.**

**Crown: How long have you been a police officer, Mr. Schwab?**

**Witness: I have been a police officer for 20 years now.**

**Crown: With your extensive time as a police officer, you would be able to determine whether someone’s story is fabricated? Is this a safe assumption?**

**Witness: I’d like to think so, yes. But you can never be 100% certain.**

**Crown:**Thank you Mr. Schwab. No further questions.

The Defence cross-examines the witness.

**Defence:** Mr. Schwab, you state that you found Casey Erickson’s statement to be credible, correct?

**Witness:** That is correct.

**Defence:** There was no doubt in your mind that **he/she** was telling the truth?

**Witness:** No, as I mentioned previously, there is no way that someone could have faked the emotions **he/she** was displaying when describing the details of the assault.

**Defence:** Are you an expert on emotions, Mr. Schwab?

**Witness:** Well, no. But I’d like to think that I could tell whether someone was faking.

**Defence:** So, basically, all we have to go on is your *opinion* as to whether **he/she** was telling the truth?

**Witness:** I suppose that’s true, however, **he/she** told me the story of the assault many times, and **his/her** story never changed.

**Defence:** Mr. Schwab, do you think that it is possible that if someone was motivated to lie that they could keep their story straight if they really wanted to?

**Witness:**  I mean, it is definitely possible, but Casey’s story just seemed so believable.

**Defence:** Exactly, Mr. Schwab, it is definitely possible. No further questions.

The Crown calls their second witness, the victim, Casey Erickson, to the stand.

**Crown:** Can you please state your name for the court?

**Witness:** My name is Casey Erickson.

**Crown:** Can you tell me your relationship with the defendant, Taylor Dunn?

**Witness:** Taylor and I were friends.

**Crown:** Thank you, and can you tell me what happened with **him/her** the night of the assault?

**Witness: He/She invited me over to his/her apartment for dinner. We ordered takeout and then when we finished eating, Mr./Ms. Dunn suggested that we watch some TV. While we were watching TV, Mr./Ms. Dunn moved close to me and put his/her hands on my lap and then began kissing me.** I told **him/her** that **he/she** was being inappropriate, but **he/she** didn’t stop. **He/she** then reached **his/her** hands into my pants and began touching me. I told **him/her** I was really uncomfortable and asked **him/her** to stop, but **he/she** continued to assault me.

**Crown:** I’m sorry, Casey, that must be hard to relive.

**Witness: Yes, thank you.**

**Crown: Are you confident that you will get justice against Taylor Dunn?**

**Witness: Yes.**

**Crown:**No further questions your Honour.

The Defence cross-examines the witness.

**Defence:** Casey, you say this interaction was unwanted correct?

**Witness:** Yes.

**Defence:** What did you do when **he/she** allegedly came onto you?

**Witness:** I asked **him/her** to stop.

**Defence:** Did you think that **he/she** understood that you were uncomfortable?

**Witness:** Yes, I think so. I told **him/her** that I wasn’t interested, but **he/she** didn’t listen.

**Defence: Is it true that you had been having casual, consensual sex with Taylor Dunn for a few months?**

**Witness: I mean, yes, that’s true. But I don’t see why that really matters.**

**Defence: Do you think that Taylor Dunn might not have viewed the interaction as unwanted because you two had been having casual sex for months?**

**Witness: Just because I was having casual sex with Taylor doesn’t mean I wanted the sexual contact that night.**

**Defence:**Are you sure you are not exaggerating the events of that night to get attention?

**Witness: No, of course not. I know that some people might not believe that something like this would happen to an Indigenous/White man/woman like myself, but I would not make up such a serious allegation.**

**Defence:** That is all your Honour. Thank you.

The Crown calls their final witness, Shauna Fisher, to the stand.

**Crown:** Hi Shauna, can you please state your full name and affiliation with the victim?

**Witness:** My name is Shauna Fisher. I’m a good friend of Casey’s.

**Crown: Shauna, did Casey confide in you about the assault?**

**Witness: Yes, he/she called me immediately after leaving Taylor’s apartment the night of the assault.**

**Crown:** Do you believe your friend and what **he/she** said happened with Taylor Dunn?

**Witness: Yes, there is no doubt. He/She wouldn’t lie to me. Casey was also very distraught when he/she called me that night. I have never seen him/her that upset before.**

**Crown:** How can you be so sure that Casey wasn't lying about the assault?

**Witness:** We have been friends for a very long time; I can tell when **he/she** is lying.

**Crown:**Thank you. No further questions.

The Defence cross-examines the witness.

**Defence:**  Shauna, you say that you believe Casey’s story, correct?

**Witness: Yes, that is correct.**

**Defence:** And you say that you can tell when Casey is lying?

**Witness: Yes.**

**Defence: So, it is true that you have witnessed Casey lying before?**

**Witness: I mean, yes... but never any serious lies. Everyone makes up lies occasionally.**

**Defence: If you have witnessed Casey lying before, how do you know that he/she wouldn’t lie about something like this?**

**Witness: I mean, I just know that he/she wouldn’t do something like that. Casey is an honest person.**

**Defence: Thank you.** Nothing further your Honour.

The Defence calls their first witness, Lucas Lovell.

**Defence:** Good morning. Could you please state your name and your affiliation with the defendant?

**Witness:** My name is Lucas Lovell, and I am a close friend of Taylor’s.

**Defence:** Mr. Lovell, your friend, **Mr./Ms.** Dunn, is being charged with sexual assault. Does this sound like something **he/she** would do?

**Witness:** I wouldn’t think so! **He’s/She’s** a great friend, and always treats **his/her** friends and family with respect. I could never see him doing something like this.

**Defence:** Why don’t you think Taylor could have committed this offence?

**Witness:**I have been friends with Taylor for many years. I have spent a ton of time with **him/her**, in groups of people and alone and **he/she** has never acted inappropriately. I just don’t think **he/she** would do something like that. Casey likely just misconstrued the situation and then felt embarrassed after. Taylor is a very friendly person! **He/She** is always laughing and talking with everyone, and **he/she** probably thought Casey was a good target.

**Defence:**Thank you, nothing further your Honour.

*The crown cross-examines the witness.*

**Crown:** Mr. Lovell, **Mr./Ms.** Dunn is a close friend of yours, correct?

**Witness:** Yes, that’s correct.

**Crown:** And do you trust **him/her** completely?

**Witness:** I do.

**Crown:** Are you familiar with the victim?

**Witness:** Well, somewhat. We live in a small town, so I’ve met **her/him** a couple of times in passing.

**Crown:** And do you trust **her/him**?

**Witness:** I don’t know **her/him** enough to trust **her/him**. **She/He** seemed nice, but I feel that **she/he** has made a mistake in this situation. Anyone who knows Taylor knows that this is a mistake and **she/he** could never do anything like this. I have never once seen **him/her** act like that with anyone. I trust **her/him** completely.

**Crown:** And so, you think this **woman/man** is making up this traumatic story?

**Witness:** I guess so. I feel really badly saying this, and if it weren’t Taylor I would be more sympathetic to **her/him**, but I just know that Taylor wouldn’t do this.

**Crown:** Seems like you might not know **her/him** quite as well as you thought. No further questions your Honour.

The Defence calls their second witness, the defendant, Taylor Dunn, to the stand.

**Defence:**Please state your name for the Court.

**Witness:**My name is Taylor Dunn.

**Defence: Please state to the court the nature of your relationship with Casey Erickson?**

**Witness: Casey and I had a sexual relationship for a few months.**

**Defence: Why do you think you were accused of this crime?**

**Witness: I don’t know, maybe he/she wants attention?** People know what kind of person I am. I really have no idea why **he/she** would accuse me of this besides to get attention or money or something.

**Defence: Can you please explain to me what happened on the night of October 17?**

**Witness:**Well, it really seemed like nothing to me. I didn’t even think about it much after **he/she** went home. We finished eating dinner and then started watching some TV on the couch. While watching TV, Casey got close to me and we began kissing, and then we both agreed to have sex. Casey never told me to stop.

**Defence: If that’s what happened, then why does Casey think you assaulted him/her?**

**Witness: I have no idea. I’m very confused by the whole situation. I would hope that he/she wouldn’t make these accusations for attention, but I guess anything is possible.**

**Defence:**No further questions your Honour.

The Crown cross-examines the witness.

**Crown:**Hello **Mr./Ms.** Dunn.

**Witness:**Hello.

**Crown:**You claim you did not sexually assault my client, correct?

**Witness:**Yes, that is correct.

**Crown: Do you believe that Casey is making up what happened between you two?**

**Witness: Well, he/she is not lying about us having relations. As I said, we kissed and had sex that night, but Casey wanted to do that just as much as I did. He/She is making up that I sexually assaulted him/her.**

**Crown:** Why would **he/she** make that up?

**Witness:** I don’t know? **He/She** might feel embarrassed about the situation? Maybe **he/she** wants money or attention? There are so many possibilities. I just know that this accusation is entirely false.

**Crown:** Or, maybe you are embarrassed that it happened so you are denying it?

**Witness:** No. I’m not lying about this. I have told the truth.

**Crown:** That is all your Honour. Thank you.

The Defence calls their final witness, Megan Baird, to the stand.

**Defence:** Hi Megan how are you today?

**Witness:** I’m good, thank you.

**Defence:** Megan, can you please state your affiliation with the Defendant?

**Witness:**  Taylor and I are good friends.

**Defence:** Where did you meet **Mr./Ms.** Dunn?

**Witness:** We got hired at a firm at around the same time as each other. We worked together for about 4 years.

**Defence:** Mrs. Baird, how do you feel about the accusations against **Mr./Ms.** Dunn today?

**Witness:** I find them very upsetting. I know Taylor would never do anything like this and it makes me so frustrated that someone could lie like this and destroy someone’s life.

**Defence: So, you think that it is very unlikely that Taylor committed this sexual assault?**

**Witness:** Absolutely! I know what type of person **he/she** is! I’ve seen the way that **he/she** treats **his/her** co-workers, clients, friends, and family.

**Defence:** Thank you, Megan. No further questions your Honour.

The Crown cross-examines the witness.

**Crown:** Megan, you say you and Taylor worked together in the past, correct?

**Witness:** Yes, that is correct.

**Crown:** Can you remember any complaints against Taylor, from the job that you both worked at?

**Witness:** Well, I mean yes. There was one co-worker who did not like Taylor for some reason. This person made an official complaint again Taylor, claiming that **he/she** had been acting inappropriate towards them.

**Crown:** What did the complaint say?

**Witness:** It said that **he/she** made derogatory comments and that **he/she** had been acting inappropriately.

**Crown:** Insulting and inappropriate behaviour? That doesn’t sound like a very respectful and trustworthy **man/woman** to me.

**Witness:** No, it wasn’t like that! That person was equally disrespectful to Taylor. It was mutual.

**Crown:** That is all your Honour.

The Crown makes their closing statement.

**Crown:**Throughout the course of this trial it has become evident that Taylor Dunn took advantage of my client, an I**ndigenous/White man/woman**, by sexually assaulting **her/him** on the evening of October 17. Although **Mr./Ms.** Dunn claims that the sex was consensual, my client made it very clear that the sex was non-consensual by repeatedly asking Taylor to stop and telling **him/her** that **he/she** was uncomfortable. I ask that you trust my client and the evidence presented to you and that you find the defendant, Taylor Dunn, guilty of sexual assault.

The Defence makes their closing statement.

**Defence: M**y client, Taylor Dunn, did not sexually assault **Mr./Ms.** Erickson. Throughout the course of this trial there were no hard facts or evidence presented that demonstrate that my client assaulted **Mr./Ms.** Erickson. Although my client did admit that **he/she** and Casey had sexual relations that night, it was entirely consensual. **Further, it is known that Taylor and Casey had been having a consensual sexual relationship for months prior to the evening of October 17.** I am hopeful that you will all agree that **Mr./Ms.** Erickson’s accusations do not seem credible. I trust that you will examine the evidence and decide that Taylor Dunn is not guilty of sexual assault.

Instructions to the Jury

**Judge:**  It is your duty to decide whether the Crown has proved **Mr./Ms.** Dunn’s guilt beyond a reasonable doubt. You have now heard all the evidence that will be called in this case. You must make your decision based on all the evidence presented to you in the courtroom and only on that evidence. You must consider the evidence and make your decision without sympathy, prejudice or fear. You must not be influenced by public opinion. Your duty as a juror is to assess the evidence impartially.

**Judge:**  **Mr./Ms.** Taylor Dunn is charged with sexual assault **(**Section 271 of the Criminal Code). You must find Taylor Dunn not guilty of sexual assault unless the Crown has proved beyond a reasonable doubt that Taylor Dunn is the person who committed the offence. Specifically, the Crown must prove the following essential element beyond a reasonable doubt:

A person commits assault when, without the consent of another person, he applies force intentionally to that other person, directly or indirectly.

Unless you are satisfied beyond a reasonable doubt that the Crown has proved all this essential element, you must find **Mr./Ms.** Dunn not guilty of sexual assault.

If you are satisfied beyond a reasonable doubt of all these essential elements, you must find **Mr./Ms.** Dunn guilty of sexual assault.
